# Novel plant–frugivore network on Mauritius is unlikely to compensate for the extinction of seed dispersers

**DOI:** 10.1038/s41467-023-36669-9

**Published:** 2023-02-23

**Authors:** Julia H. Heinen, F. B. Vincent Florens, Cláudia Baider, Julian P. Hume, W. Daniel Kissling, Robert J. Whittaker, Carsten Rahbek, Michael K. Borregaard

**Affiliations:** 1 Center for Macroecology, Evolution and Climate, Globe Institute, University of Copenhagen, 2100 Copenhagen, Denmark; 2 Department of Biosciences and Ocean Studies, TIBEC Pole of Research, University of Mauritius, Réduit, 80837 Mauritius; 3 The Mauritius Herbarium, Agricultural Services, Ministry of Agro-Industry and Food Security, Réduit, 80835 Mauritius; 4 Department of Life Sciences, Natural History Museum, Tring, HP23 6AP UK; 5 Institute for Biodiversity and Ecosystem Dynamics, University of Amsterdam, 1090 GE Amsterdam, The Netherlands; 6 School of Geography and the Environment, University of Oxford, Oxford, OX1 3QY UK; 7 Center for Global Mountain Biodiversity, Globe Institute, University of Copenhagen, Universitetsparken 15, 2100 Copenhagen, Denmark; 8 Institute of Ecology, Peking University, 100871 Beijing, China; 9 Danish Institute for Advanced Study, University of Southern Denmark, 5230 Odense M, Denmark

**Keywords:** Biogeography, Community ecology, Invasive species, Ecological networks, Conservation biology

## Abstract

Insular communities are particularly vulnerable to anthropogenic extinctions and introductions. Changes in composition of island frugivore communities may affect seed dispersal within the native plant community, risking ecological shifts and ultimately co-extinction cascades. Introduced species could potentially mitigate these risks by replacing ecological functions of extinct species, but conclusive evidence is lacking. Here, we investigate changes in plant–frugivore interactions involving frugivorous birds, mammals and reptiles in Mauritius, an oceanic island with an exceptionally well-specified frugivore community and well-described species introduction history. We demonstrate substantial losses of binary interaction partnerships (at the species level) resulting from native species extinctions, but also gains of equal numbers of novel interactions with introduced species, potentially supporting the idea that non-native species might compensate for lost seed dispersal. However, closer investigation of animal seed handling behaviour reveals that most interactions with seed dispersers are replaced by ecologically different interactions with seed predators. Therefore, restoration of seed dispersal functionality in this novel plant–frugivore community is unlikely.

## Abstract (web summary)

Many plant species depend on frugivores for seed dispersal. Here, the authors investigate plant-frugivore networks in Mauritius, finding that the new interactions gained from the arrival of non-native seed predators are unlikely to compensate for the extinction of seed dispersers.

## Introduction

Remote oceanic islands are known for their high proportions of endemic species. Many of these possess characteristic island syndromes, such as animals with reduced or lost flight capacity and gigantism. This makes them particularly vulnerable to pressure from introduced, invasive species and elevates their extinction risk, making islands global hotspots of species extinction (totaling, e.g., close to 90% of all extinct birds in 3.5% of Earth’s area^1^). Large and flightless birds such as the iconic dodo, *Raphus cucculatus*, have gone extinct at a disproportionate rate to other species^2–5^, whereas introductions have been dominated by a narrow selection of highly opportunistic species, e.g., black rats *Rattus rattus* and pigs *Sus scrofa* accompanying early explorers^6,7^. These processes have shifted community composition across islands in recent history^3,5,8–10^, in turn altering interaction networks among species^11,12^. Such disruption of ecological dynamics may change eco-evolutionary feedbacks and ultimately lead to co-extinction cascades among remaining native species^13–16^.

Animal-dispersed plants are at risk of co-extinction when frugivores that disperse their seeds disappear^16–20^. Frugivore-mediated seed dispersal is a crucial mutualistic interaction for many plants, as the transport of seeds to new sites drives metapopulation dynamics, decreases intra-specific competition and lowers predation pressure on seedlings, in turn increasing germination and recruitment success^21–25^. The loss of many large frugivores from island communities in particular puts native large-seeded plants at risk of extinction, as the remaining small native frugivores cannot swallow and/or disperse the largest seeds, like those adapted to dispersal by large extinct pigeons in Tonga^3,15,18,21,26,27^.

If unmitigated, such secondary ecological effects pose major challenges for the long-term sustainability of island ecosystems. However, a number of recent papers have argued that the anthropogenic introductions of new frugivores to an island might save native plants from dispersal-loss extinction by functionally replacing the extinct frugivores as seed dispersers, such as suggested for the Rock Dove *Columba livia* in the Pacific^28–30^. A less positive picture is provided by a study of seed dispersal networks in Oahu Island (Hawaii) in which it was shown that while non-native birds do disperse native plant species, fewer than 7% of interactions involved native plants^31^. This serves to highlight that in order to predict the long-term consequences of native frugivore extinctions for native plants, studies are required that combine multiple aspects of interacting species in island communities, incorporating both extinctions and introductions and detailing the full functional ecological networks of interacting groups. This goal, however, has been hard to achieve given the paucity of comprehensive ecological data on most islands (e.g., species occurrences, functional traits, interactions, behavior). Previous studies have rarely been able to consider complete communities of interacting species^32,33^ or only looked at either extinctions or introductions^3,9^.

Here, we combine data from historical and paleontological records, museum and herbarium specimens and ongoing conservation programs, for all known frugivores on the Indian Ocean island of Mauritius. The dataset details all native, introduced, and extinct birds, mammals, and reptiles that feed on native fruits, along with their native food plants, their functional traits related to seed dispersal, and the known binary interactions between them. As our focus lies on the fate of zoochorous native plants, because these depend on the available seed dispersers on the island, non-native plants in the frugivore diets are not included because those do not directly affect this. The dataset covers more than four centuries, including both the pre- and post-human disturbance communities.

Mauritius is exceptionally well documented and is thus an ideal system for a comprehensive study of the effects of extinctions and introductions of frugivores on the potential seed dispersal services provided to the native flora. It is famous for the extinction of many iconic fruit-eating species, including the Dodo *Raphus cucullatus* and two giant tortoises *Cylindraspis* spp.^34^. After relatively recent permanent colonization, starting with the Dutch in AD 1638, only about 4.4% of the original native habitats remain^35^, and most native species are either extinct, severely reduced in abundance and range, or under threat from introduced species^34,36^. There has been a marked shift in community composition of frugivores (birds, mammals, reptiles), with only 15 of 26 native species surviving and 14 non-natives having become established in the wild^3,34^.

Our central question: does the novel frugivore–native plant network on Mauritius compensate for the extinction of seed dispersers? is based on three key component hypotheses. Here, we address each of these three hypotheses in turn:

1. Functional traits of animals affect their interactions, e.g., larger-bodied species are able to disperse larger seeds. This is generally expected from ecological theory, as smaller frugivores are generally incapable of swallowing large seeds, have smaller gut capacity, shorter gut retention times, and are less likely to transport seeds further away than large frugivores^3,11,18,22,26,27,32^.
2. Species loss in itself has led to substantial degradation of the interaction network between Mauritian frugivores and the native fruiting plant community. The loss of interactions caused by extinctions has been observed previously in several different island systems^31^ and for it not to result in interaction network degradation, needs to be offset by novel interactions between introduced frugivores and native Mauritian plants.
3. The introduced frugivores interact with fruits in a similar way as native frugivores. There are differences in seed handling and fruit consumption behavior by different frugivores^13,22,37^, such as seed destruction or dispersal^38,39^, which can have strong consequences for interaction outcomes at different demographic stages of plants^40^. If, as suggested by studies elsewhere, introduced frugivores might also prevent dispersal-loss extinction of native plants on Mauritius, novel interactions with introduced frugivores need to result in the same functional outcome as the lost interactions to achieve effective replacement of ecological function^13,37^.

Here we demonstrate that native frugivore extinctions have caused substantial losses of interaction partnerships that appear to be compensated for by gains of similar numbers of novel (binary) interactions with introduced frugivores. However, seed handling behavior by frugivores has shifted from seed dispersers to more seed predators, making the apparent restoration of seed dispersal functionality in the novel plant–frugivore community unlikely.

## Results

### Body mass—seed size relationships

**Fig. 1:**
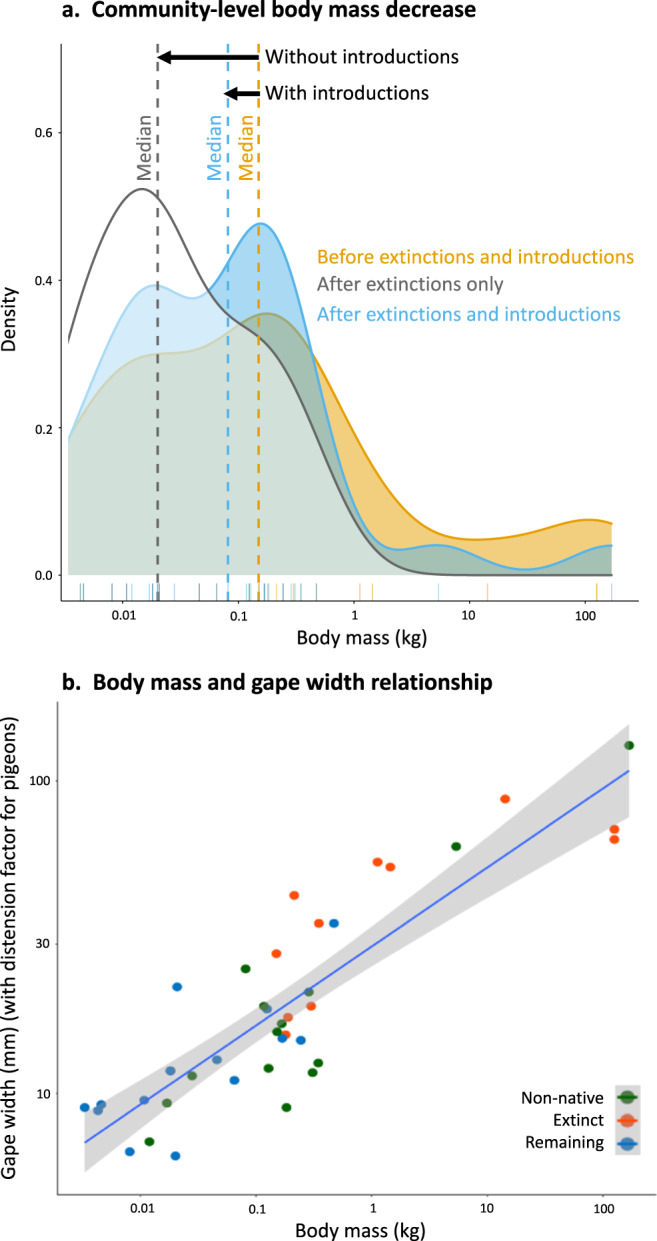
Community-level body mass decrease on Mauritius and the relationship between body mass and gape with. **a** Community-level changes in body mass of frugivores in Mauritius, before and after extinctions and introductions of birds, mammals and reptiles. The decrease in median body mass caused by the extinction of many large frugivores (community median before 159 g to 20 g after, without introduced frugivores) appears to be partially compensated for by the introduction of non-native frugivores (community median before 159 g to 85 g after, with introduced frugivores). **b** Frugivores on Mauritius with larger body mass have larger gape sizes (as width of bill or mouth) than smaller species, possibly enabling them to swallow and disperse larger seeds (linear regression in R, all frugivores, *n* = 40 species, *p* = 2^−16^, adj. *R*^2^ = 0.76, df = 38, standard error in gray, pigeon gapes distended 1.9 times according to Meehan et al.^26^).

Both extinctions and introductions have been pronounced in Mauritius, with 11 (seven birds, two mammals, three reptiles) of the 26 (14 birds, three mammals, ten reptiles) original frugivore species going extinct, while 14 (ten birds, four mammals) new frugivores have been introduced. In particular, many of the large frugivores, e.g., two giant tortoises *Cylindraspis* spp. and a giant skink *Leiolopisma mauritianus* (Fig. 1), able to swallow and disperse the largest seeds, have gone extinct. Extinctions have caused a community-level reduction of median frugivore body mass of 87% (Fig. 1a, after extinctions only, median 159 to 20 g, interquartile range 319 to 85 g) of that of the original native frugivore community (presence-absence). At the same time, many small frugivores (and only two large frugivores, the pig *Sus scrofa* and macaque *Macaca fascicularis*) have been introduced, resulting in an overall median body mass reduction of 48% (from 159 to 82 g, with the interquartile range decreasing from 319 to 165 g; Fig. 1a, after extinctions and introductions, presence-absence). The difference among body mass distributions of extinct, introduced, and remaining frugivore species was highly significant (ANOVA, *F*_37,2_ = 10.343, *p* = 0.00027; Kruskal–Wallis *Χ*^2^ = 16.477, *p* = 0.00026, see Supplementary Fig. 1). The greatest difference was between extinct and persisting native species, whereas extinct and introduced species had more similar body mass (for Tukey post hoc see Supplementary Table 1; and without large introduced species Supplementary Table 2).

**Fig. 2:**
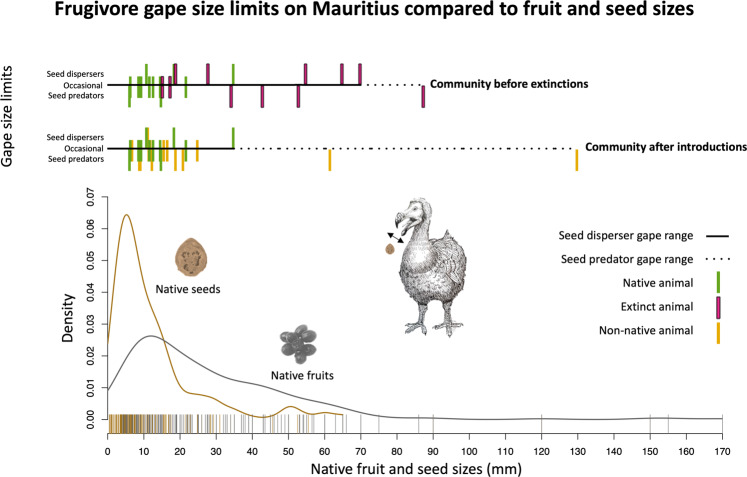
Frugivore gape size limits on Mauritius compared to native fruit and seed sizes. The extinction of large frugivores on Mauritius has led to a reduced gape size limitation for seed dispersers and at the same time, the introduction of non-native frugivores had led to an increased gape size for seed predators (maximum fruit and seed dimensions, *n* = 263 plant species, all frugivores, *n* = 40 animal species, pigeon gapes distended 1.9 times according to Meehan et al.^26^). Justifications for seed handling categorization in Supplementary Data 3. Plant names in Supplementary Data 1. Drawing by J.P.H.

There is a strong linear relationship between body mass and gape width (Fig. 1b, all frugivores, *n* = 40, df = 38, *p* = 2^−16^, adj. *R*^2^ = 0.76), indicating that extinct large animal species also had large gapes and could likely disperse large seeds. The maximum community-level gape size for native frugivores classified as general seed dispersers on Mauritius (justified in Supplementary Data 3) has been reduced by 50% through extinctions (Fig. 2), as the extinct saddleback giant tortoise *Cylindraspis triserrata* had a gape width of 70 mm, while the largest extant species, the Mauritian flying fox *Pteropus niger* has a gape width of 35 mm. At the same time, the maximum gape width of frugivores classified as general seed predators on Mauritius (justified in Supplementary Data 3) has increased by 49%, from 87.5 mm for the now extinct Dodo *Raphus cucullatus* to 130 mm for the introduced feral pig *Sus scrofa*. Currently, 28% (74) of the native fruits and 7% (19) of the native seeds are now left larger than the maximum gape size of the extant seed disperser community, whereas this used to be only 3% (7) for fruits and 1% (3) for seeds (Fig. 2, native Mauritian zoochorous plant species, *n* = 263, max fruit and seed size dimensions).

### Ecological shifts in plant–frugivore interactions

**Fig. 3:**
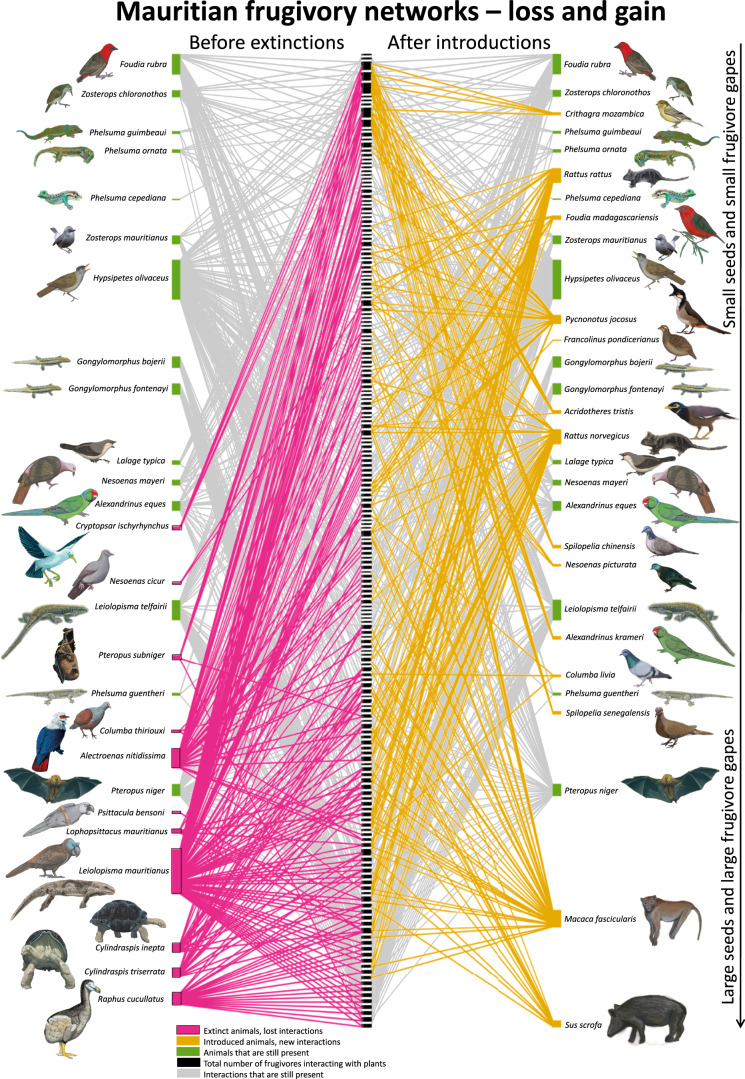
Loss and gain of interactions in Mauritian frugivory networks before extinctions and after introductions. Interaction network between frugivores and native fleshy-fruited plants on Mauritius, before and after frugivore extinctions and introductions. Pink lines indicate lost interactions and yellow lines indicate gained interactions. Plant names in Supplementary Data 2. Version with distinction between data origin in Supplementary Fig. 5. Drawings by J.P.H. and J.H.H.

Despite non-random changes in functional traits (e.g., body mass and gape size, Figs. 1 and 2) in the frugivore community there appears to be a similar loss and gain of plant–frugivore interactions (including swallowed and carried fruits, Fig. 3). Extinctions led to the loss of 203 of the originally registered 558 fruit-feeding interactions with 119 out of 191 native fleshy-fruited plants, whereas introductions led to the gain of 173 novel fruit-feeding interactions with 74 native plants (leading to a net loss of only 5% of the original number) (Fig. 3). The similarity of numbers seems to indicate no significant shift in the plant–frugivore interaction network, if differences in seed handling behavior are not considered.

### Seed handling

**Fig. 4:**
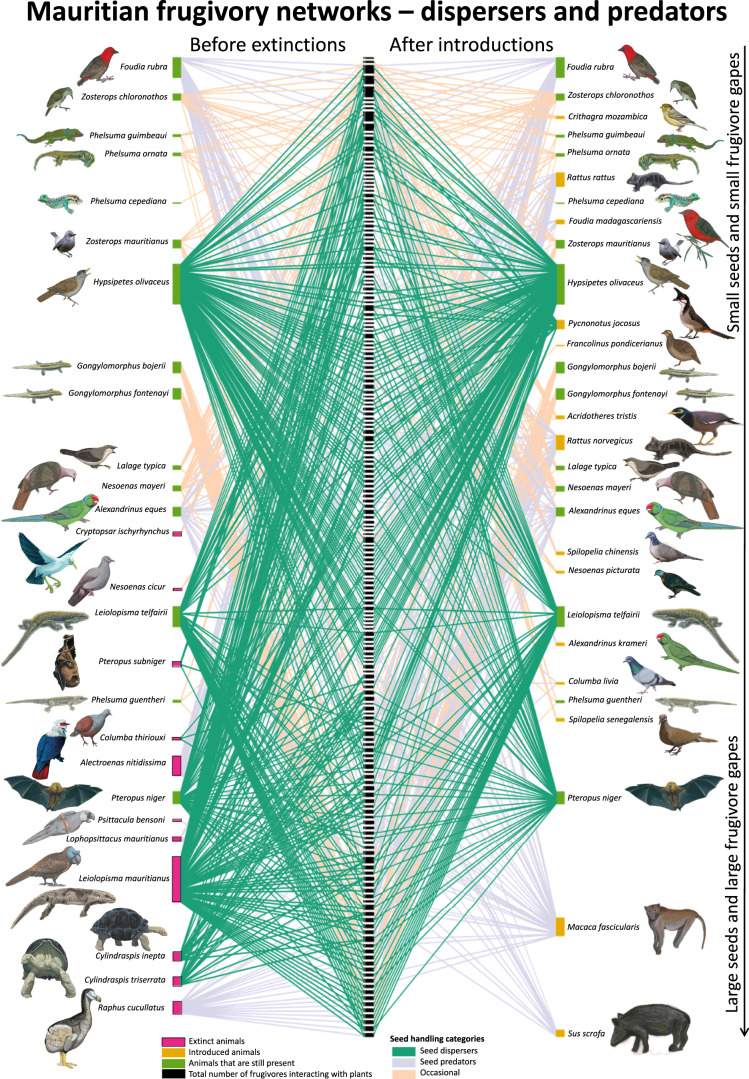
Interactions with seed dispersers in Mauritian frugivory networks before extinctions and after introductions. Interaction network between frugivores and native fleshy-fruited plants on Mauritius, before and after frugivore extinctions and introductions. Green lines indicate interactions with seed dispersers. Justifications for seed handling categorization in Supplementary Data 3. Plant names in Supplementary Data 2. Version with distinction between data origin in Supplementary Fig. 6. Drawings by J.P.H. and J.H.H.

Many interactions with extinct seed dispersers (five species) have been replaced by novel interactions with introduced seed predators (seven species) (Fig. 4, seed handling justifications in Supplementary Data 3). Although all frugivores have the potential to contribute to seed dispersal to varying degrees, those that generally predate on seeds are functionally different because they are likely to contribute much less to the recruitment success of native plants. For example, the percentage of seeds destroyed after handling can be up to 100% for macaques *Macaca fascicularis* on Mauritius because they pick unripe fruits before seeds become viable^41–43^, and studies from other locations have reported percentages of 86% for pigs (*Sus scrofa*^44^) and ~65% for rats (*Rattus rattus*^45^). The replacement of extinct interactions with novel ones has resulted in a 36% (304–196) reduction in seed dispersal interactions and an increase of 54% (126–194) of mostly seed-destructive interactions. Extinctions of seed dispersers have left many plants with fewer seed dispersal interactions, in particular with fewer large frugivores potentially able to disperse the largest seeds (Figs. 1 and 2). Two of the three persisting native seed dispersers, Mauritian flying fox *Pteropus niger* and Mauritian bulbul *Hypsipetes olivaceus*, now seem to fulfill disproportionally large ecological roles (for flying fox see ref. ^46^), together accounting for 90% (176–196) of those current interactions with native plants that likely lead to seed dispersal (Fig. 4). The extinction of any of the remaining threatened seed dispersers, including Telfair’s skink *Leiolopisma telfairii*, would leave many native Mauritian plants without frugivore-mediated seed dispersal (Fig. 4), as is already the case in some locations^47^, negatively impacting their recruitment success and population persistence.

### Patterns of plant interaction loss

**Fig. 5:**
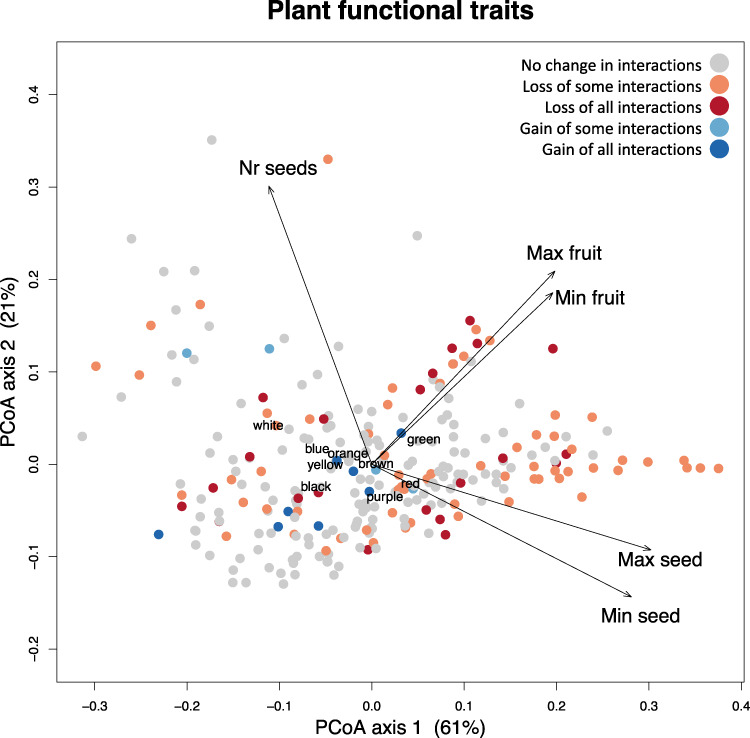
Functional trait space of native Mauritian fleshy-fruited plants. Functional trait space of all native fleshy-fruited plants on Mauritius by means of a PCoA (*n* = 263 plant species, PCoA, Gower distance, Supplementary Fig. 2, 25% missing data imputed with 80% accuracy, Supplementary Table 3). Arrow directions indicate the relationships between maximum and minimum size dimensions of seeds and fruits, and the number of seeds per fruit. The fruit color positions are indicated with their names. Points indicate plant species, and their colors indicate changes in known plant–frugivore interactions due to extinctions and introductions of frugivores. There was no change in known fruit-feeding interactions (gray), loss of one or more interactions (orange), loss of all known interactions with the plant (red), gain of some known interactions (light blue) or gain of all known interactions (dark blue). Notice the loss of interactions throughout the trait space and the gain of interactions with small seeds and fruits only. Plant names and axis values in Supplementary Data 1.

Contrary to expectations, the loss of interactions due to non-random frugivore extinctions (Figs. 1 and 2, see also ref. ^3^) appears to have happened evenly throughout the functional trait space of the native plants, with no particular pattern characterizing the species that have lost most interactions (Fig. 5, PCoA, maximum and minimum fruit and seed size, seeds per fruit, and fruit color, *n* = 263, 25% imputed with missForest using phylogeny, 82.23% variation explained in two axis). In contrast, animal introductions clearly favored interactions with small-seeded and small-fruited plants, except the feral pig *Sus scrofa*, which generally predates seeds in Mauritius.

**Fig. 6:**
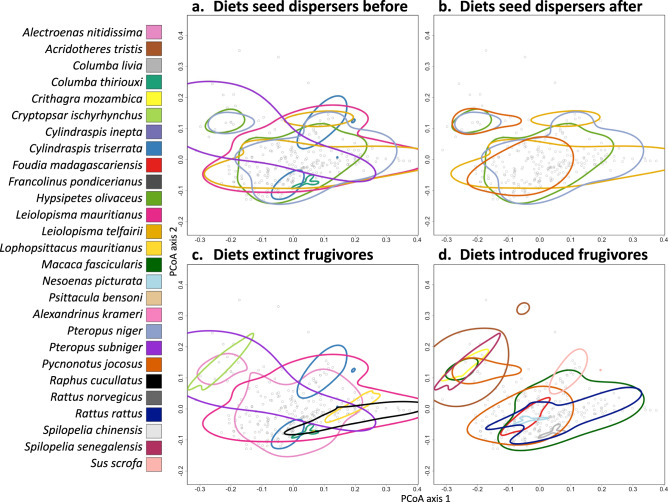
Diets of frugivores on Mauritius within the functional trait space of native plants. Known fruit-feeding interactions between frugivores and native Mauritian plants within the functional trait space of the plants (*n* = 263 plant species, PCoA, Gower distance, variation explained by axis 1 = 61.33%, axis 2 = 20.9%, Supplementary Fig. 2, 25% missing data imputed with 80% accuracy, Supplementary Table 3). The interactions of each animal are indicated by a circle around the 95% kernel density estimate. First two panels show seed dispersal interactions before (**a**) and after (**b**) extinctions and introductions to discuss the loss of interactions throughout the network, despite introductions. Second two panels show extinct frugivores (**c**) and introduced frugivores (**d**) to discuss functional replacement. Plots per species in Supplementary Fig. 4. Justifications for seed handling categorization in Supplementary Data 3. Plant names and axis values in Supplementary Data 1.

Most seed dispersers eat fruits with a wide variety of traits and have substantial dietary overlap (Fig. 6a, b, including swallowed and carried fruits). However, the two extinct giant tortoises appear to have eaten mostly very large fruits^48^ (Fig. 6a). In general, most of the trait space of the previous community is still covered by potential dispersers, but by fewer species (Fig. 6a, b). The common and locally dominant Red-whiskered bulbul (*Pycnonotus jocosus*) is the only introduced seed disperser, but appears not to eat the largest seeds and fruits (Fig. 6b, d and Supplementary Fig. 4, points on right side not included), and thus cannot compensate for the dispersal of large-fruited plants by extinct seed dispersers, such as the giant tortoises. Large-fruited plants (Figs. 5 and 6, top right points) are currently only dispersed by the remaining native frugivores, all of which are threatened with extinction^34,49^.

To assess whether the introduced frugivores could replace the seed dispersal function of extinct frugivores, we compared the whole range of native fruit traits within their diets (Fig. 6c, d) rather than only considering the identified interactions, thereby overcoming any unobserved interactions or potential imbalance in data collection. Several of the extinct species appear to have been highly selective. The adult Dodo *Raphus cucullatus* and giant tortoises *Cylindraspis inepta* and *C. triserrata*, and Broad-billed parrot *Lophopsittacus mauritianus* all likely preferred large seeds and fruits, whereas the Mauritius starling *Cryptopsar ischyrhynchus* most likely preferred small fruits like the other Mascarene starlings^50–52^. Together, the diets of the introduced frugivores (Fig. 6d) cover a large range of plant traits, with some avoidance of the largest fruits (top right). The total functional volume of plant traits covered by at least one frugivore has not diminished in size, though large areas of this trait space are today covered only by a small number of frugivores that feed on a wide variety of native Mauritian plants.

## Discussion

There have been substantial losses of plant–frugivore interactions on Mauritius as a consequence of animal extinctions, leading to the formation of a novel seed-disperser network with native plants, potentially leading to different ecological dynamics. The gain of a similar number of novel plant–frugivore interactions probably did not compensate for potential losses in seed dispersal, because the key introduced frugivores are often seed predators that generally destroy a large proportion of handled seeds (e.g., up to 100% for macaques^41–43^, 86% for pigs^44^ and ~65% for black rats^45^, Supplementary Data 3). In addition, the maximum size of seeds that can be swallowed by seed dispersers has decreased, while that of seed predators has increased, which could negatively affect large-seeded plant recruitment. The only introduced seed disperser, the red-whiskered bulbul, is not known to disperse the largest-fruited or largest-seeded plants, leaving these dependent on a few remaining native frugivores that are threatened with extinction^34,49^. Therefore, the inferred negative impact on plant germination, recruitment success, gene flow, habitat connectivity, source-sink dynamics and local rescue effects^20–27^ potentially leaves the native plant community vulnerable to severe declines in abundance and secondary extinctions. Even though we do not show this here directly, there have been several empirical studies linking lower plant recruitment with frugivore extinctions^21,27^, including in the Mascarenes^53^.

The loss of large frugivores has caused a community-level reduction of the mean body mass, a pattern also observed for frugivores globally^3^, in spite of the introduction of a few large frugivores. This adds further complications for seed dispersal because small frugivores can only swallow or carry small seeds, have a small gut capacity, short gut retention times, and are generally less efficient at long-distance dispersal^3,11,18,22,26,27^. Specifically, the size of seeds that can be ingested decreased for seed dispersers and increased for seed predators. This risks the loss of gut-passage seed dispersal for large-seeded plants and possibly increases seed predation. This supports the first hypothesis that the non-random extinction of many large-bodied species could affect the ability of the frugivore community to disperse larger seeds. Though large seed predators such as pigs may occasionally disperse viable seeds (through interactions that can vary along a continuum of mutualism and antagonism^40^, and such rare dispersal events may in principle be enough to prevent plant extinctions^13^, the functional outcome of seed predation is substantially different from that of primary seed dispersers.

Interactions with frugivores were lost evenly throughout plant functional trait space, highlighting the broad vulnerability of native fleshy-fruited plants to the loss of seed dispersal services. The main exception was a tendency for the largest-fruited plants to lose more interaction partnerships, a pattern also found in other island systems^3,26,27,32^, whereas new interactions with non-native frugivores mainly occurred with small-seeded and small-fruited plants. Thus overall, this suggests that the novel plant–frugivore network on Mauritius may not compensate for the extinction of seed dispersers.

The remaining plant–frugivore interactions on Mauritius are influenced by factors related to habitat disturbance, reduced abundance, interaction frequency, introduced species behavior, variation in seed handling per interaction, fruit and seed morphology that influences seed handling, plant life cycle and degree of dependency on frugivore-mediated seed dispersal. At present, the severely reduced, fragmented, and disturbed natural habitats in Mauritius make it difficult for some plants and frugivores to interact by reducing range overlap^34^ and causing local extinctions^47^. Other influencing factors are reduced encounter rates between species due to their rarity^13,54^, reduced fruit set because of nutrient competition with invasive plants^55,56^, destruction of fruits and flowers by monkeys and rats before they ripen^41–43,47^, and possibly reduced pollination due to pesticide use. The many intensive conservation efforts in Mauritius have been successful in increasing the population sizes of critically endangered plants and animals, weeding out invasive plants and reducing invasive animal densities^57^. This is a promising start but raises the question whether it is possible to restore the functionality of frugivore-mediated seed dispersal interactions, even under ideal (but unrealistic) circumstances that would allow all current Mauritian species to co-occur and interact again in functionally significant abundances^13^.

The role of the non-native red-whiskered bulbul as seed disperser of native plants demonstrates that it is possible for introduced species to fulfill this ecological role to some extent. However, this benefit may be outweighed by lesser-known negative influences on the native ecosystem^58,59^, such as the facilitation of dispersal of invasive plants^59,60^ which are not considered in this study. The same risk potentially applies to the introduction of non-native Aldabra giant tortoise *Aldabrachelys gigantea* and radiated tortoise *Astrochelys radiata* (not included in our data because they are not naturalized) as replacement of the two extinct giant tortoises (*Cylindraspis* spp.) to rewild several Mauritian islets, as the Aldabran species is known to disperse large native seeds^29,61^.

A key aspect that is hard to address empirically is the spatial context of species interactions^13^, as most remaining Mauritian plants and their frugivores are rare and exhibit greatly reduced areas of co-occurrence^34,54^. A notable exception being the ubiquitous flying fox which is capable of long-distance flights across the fragmented landscape^62,63^, but which nevertheless faces elevated extinction risk^49^. It is thus likely that many interactions are in practice already either functionally extinct or at high risk of becoming so.

This raises the question whether we can expect to see cascading effects of co-extinction of plants in the future. Compared with vertebrates, fewer plants are known to have gone extinct^64^ (10.9%), either because plant extinctions are difficult to prove or because there have not been many^1,65^. However, Mauritius currently holds the second highest rate of native trees threatened with extinction worldwide^66^. The number of successful interactions required for population persistence and prevention of extinction is different for each plant species. Some Mauritian plants can get very old, with a slow generation time, and their populations may be able to persist with very few successful frugivore-mediated dispersal events over their lifetime. Since current species abundances and range-overlap change rapidly due to conservation projects and invasive species, current interaction frequencies would not reflect interaction patterns at the scale of plant generation times, making it difficult to assess potential compensatory effects of changes in interaction frequency and plant extinction risk. The Mauritian plants also have a tendency to form seedling banks that can pause growth until conditions are suitable^67^ (e.g., ebonies—*Diospyros* spp.). Dependency on frugivores for dispersal of seeds is not always obligate for survival, such as for *Pandanus* species that have fleshy fruits that can also be carried by water and sometimes reproduce vegetatively^13,68^. Not all native Mauritian plants are endemic^64^ (40% angiosperms endemic), inferring that co-extinction cascades may be local rather than global.

Direct negative changes for native plant reproductive success on Mauritius as a consequence of frugivore extinctions and introductions are inferred from our results, but supported by several empirical studies on Mauritius^42,53,59^. The Janzen-Connell effect has been experimentally investigated on Mauritius for *Syzygium mamillatum* (Myrtaceae), which shows strong negative effects of proximity to maternal trees on growth and survival of seedlings^69^, indicating the importance of seed dispersers for their recruitment success. On the nearby island of Réunion, which has a similar ecological history, the loss of frugivores resulted in fruit-flesh persistence that strongly inhibited seed germination^70^. Introduced frugivores have been found to prevent development of viable seeds of several native plants by destroying unripe fruits (macaques^41–43^) and germination patterns of non-native plants benefitted from gut passage, facilitating their spread (e.g., by red-whiskered bulbuls^59^). After extinctions, seeds from the “Dodo tree” (tambalacoque, *Sideroxylon grandiflorum*, Sapotaceae) that were (probably inaccurately) thought to have co-evolved, are still able to germinate without abrasion in a strong muscular crop^41,71^. Overall, however, the current recruitment of native large-seeded plants in one of the best-preserved Mauritian forests is low^53^. Hence, any disturbances in the plant−frugivore interaction network run the risk of influencing eco-evolutionary processes and a wide range of other interactions with unknown effects. For conservation efforts to improve frugivore-mediated seed dispersal of Mauritian plants, it may be beneficial to consider not only fruit-feeding interactions, but also the functional outcomes of animal seed handling behavior, for both native and non-native frugivores.

## Methods

### Plant database construction

A database of Mauritian plants and their functional traits was compiled for this study based on the most recent Mauritian Flora^72^, expert knowledge, herbarium measurements and field observations done by co-authors (CB and FBVF). The inclusion criteria for plants were twofold: (1) they needed to offer a reward for frugivores in the form of fleshy fruit or a fleshy aril for their seed dispersal method to be considered (endo)zoochorous and (2) they needed to be a native species because these have co-occurred with native frugivores in past and present. This resulted in the inclusion of 263 of the 691 (38%) known native Mauritian flowering plants^64^ (Supplementary Data 1). Family names follow the Angiosperm Phylogeny Group system (APG) and species names follow those in the Flore des Mascareignes^72^, updated according to more recent literature by CB from the Mauritius Herbarium for this study (Supplementary Data 1 and 2).

The functional traits of plants that were used are the maximum and minimum dimensions of seeds and fruits, fruit color and the number of seeds per fruit, because these traits are relevant for seed dispersal^73,74^. Fruit color was categorized into nine basic color terms (black, blue, brown, green, orange, purple, red, white, and yellow) according to the method of Dominy et al.^75^. The trait data was based on measurements from fresh fruits and seeds (permit NP 46 V8 National Parks and Conservation Services) or re-hydrated herbarium material (Mauritius Herbarium and Kew Herbarium) and species descriptions in the Flore des Mascareignes^72^. The number of individuals measured varied with fruit availability because many native plants are very rare^64^ and only ripe fruits could be measured, one for extremely rare plants to several hundred for plants that produce ripe fruit frequently (resulting in good average sizes across regions).

Due to the rarity of many native plants, difficult access, low fruit-set, irregular phenology, and frequent fruit destruction before ripening^41–43^, it was impossible to obtain all trait data for each of the 263 plant species. This resulted in a trait database for plants that is 75% complete, which reflects the current state of knowledge, based on decades of intensive research efforts (e.g., Mauritius Herbarium, University of Mauritius, Kew Herbarium, Flora of Mauritius, Index of Mauritian flora, historical records). The missing plant trait data (25%) were imputed by means of a random forest model (R v4.1.1, missForest^76^, 80% accuracy^77^, Supplementary Table 3) that used phylogenetic relatedness^78^ by incorporating the first four eigenvectors as traits in the model (V.PhyloMaker^79^, build node 1, scenario 3, PVR^80^, explaining 22.1% of variation). To increase imputation performance, we trained the model on a larger dataset by attaching and later removing a dataset with 1537 different plant species with the same functional traits, from 111 islands across 20 archipelagos worldwide (unpub. data, project led by Donald R. Drake, Kim R. McConkey and JHH). Missing fruit colors (39%) were filled in with the most common fruit color of genus or family.

### Frugivore database construction

A database of all native, extinct and introduced frugivorous birds, mammals and reptiles in Mauritius has been compiled from the literature^34,81,82^. Species names follow the most recent accepted taxonomy. We excluded migratory and vagrant species. We included species that eat any amount of fruit. We determined frugivory (reasoning in Supplementary Data 3^81–127^) based on observations, diet database records^82,127^, and extensive literature searches (e.g., google scholar search of species name and “fruit” or “berry”) of the species themselves or of close relatives that are morphologically similar and mainly occur in the same region (for extinct species).

In order to represent the general differences in functional outcomes for plants caused by differences in seed handling, we distinguished three types of fruit-eating animals: those that are generally seed dispersers or generally seed predators for native Mauritian plants and those that only occasionally handle native seeds on Mauritius with unclear or mixed outcomes (detailed literature-based^13,34,42,44–46,48,51,52,71,81–126^ justifications in Supplementary Data 3). Seed dispersers are defined as species for which there are (literature) observations of seed dispersal, e.g., seeds in feces or mouth ejecta or physical carrying, and species that have a morphology that does not promote seed destruction, and have closely related or similar species that are known to generally disperse seeds. Seed predators are defined as animals with particular morphology that generally promotes the destruction of seeds, such as a strong bill or muscular crop, and species of which there are (literature) observations of frequent destruction of seeds of several plant species, and animals that have close relatives that meet these criteria that are morphologically similar and mainly occur in the same region. Species that occasionally handle native Mauritian seeds are those species that either do not feed on fruits frequently, or do not include a lot of fruits in their diet^127^ (<20% fruits in diet) or that do a mixture of seed dispersal and predation. These are the cases where it is less clearly defined. Literature-based^13,34,42,44–46,48,51,52,71,81–126^ justifications in Supplementary Data 3.

We included the functional traits body mass and gape width because these are relevant for the size, quantity, and distance that seeds can be dispersed^11^. The mean body mass data (across sexes) was obtained from pre-existing databases^3,128^ or calculated based on relationships with other morphometrics according to the methods of Heinen et al.^3^. The gape width measurements were defined as the distance between the commissural points of bill, beak or mouth for birds, reptiles, and mammals. They were taken from museum skins and spirit specimens (NHM Tring, NHM London, NHM Mauritius, ZM Copenhagen, La Vanille Nature park) and from bats (*Pteropus niger*, *n* = 26, Casela Nature Parks, permit NP 46 V8) and tortoises (*Aldabrachelys gigantea*, beak width and curved carapace length linear regression in R, beak width cm = 1.6682 + 0.061523 * CCL cm, *n* = 50, *p* = 6^−19^, adj. *R*^2^ = 0.81, La Vanille Nature Park, permit NP 46 V8). If this was not available or in the case of extinct species, literature was used to calculate body mass according to the methods of Heinen et al.^3^. Gape width for extinct species was measured on skin specimens (Mauritius blue pigeon, *Alectroenas nitidissima*, NHM Mauritius), subfossil bones as jaw width^51,52,99,129^ (Hume Unpubl. data, NHM Tring, NHM London, NHM Oxford, NHM Mauritius), or calculated by means of the relationship between snout vent length and jaw width in *Gekkos* and *Phelsumas* (*n* = 11, linear regression in R, jaw width mm = 1.093968 + 0.158324 * SVL mm, *p* = 3^−5^, adj. *R*^2^ = 0.97, ZM Copenhagen), or calculated from the relationship between gape size and other morphometrics in a similar species. For pigeons (incl. Dodo) we used the 1.9 times distended gape sizes by calculation according to the average used in Meehan at al.^26^ to account for jaw expansion.

### Interaction database construction

Plant–frugivore interaction data was compiled from historical records spanning approximately four centuries, and contemporary literature, observations in zoos (*n* = 2), and the field during several research and conservation projects that intensified over the past five decades (Supplementary Data 3^13,34,42,44–46,48,51,52,71,81–126^ and personal observations by CB, FBVF, JPH, JHH, Pierre Baissac, Prishnee Bissessur, Nik Cole and Dennis M. Hansen). Due to the relatively recent split^130^ between *Phelsuma guimbeaui* and *P. rosagularis*, and the resulting lack of specific interaction data for the new species, the latter species is here represented by the former.

We distinguished between interaction data of two different origins, direct observations (25%) (e.g., same species in Mauritius), and derived interactions (75%) (e.g., closely related species or same species in different locations in the Indian Ocean) (Supplementary Data 3 and Supplementary Figs. 3–6). Direct observations were those of a frugivore (extinct, introduced or remaining) eating a fruit native to Mauritius while on Mauritius. Derived interactions could be (1) observed in another location between species that both also occur on Mauritius (e.g., invasive rock pigeons), (2) or between a closely related and morphologically similar frugivore on another nearby island, eating a fruit that is also native to Mauritius (e.g., for blue pigeons and bulbuls of Indian Ocean region), (3) or an account of an observation that is less clearly defined (e.g., *Ficus*, *Diospyros* or *Mimusops* species, from giant tortoise eating “apple-like fruit”). An assessment of the sensitivity of our results to differences in data origin can be seen in Supplementary Figs. 3–6. The data generally follows a similar pattern, especially for introduced species, but cannot be interpreted separately due to the nature of extinct species observations being often less specific and harder to verify.

Our interaction data provide the most complete overview of the current state of knowledge, based on decades of research efforts and historical records, but may not contain all possible interactions and does not focus on non-native plants that may be eaten as well. Due to the large amount of available data, potentially unobserved interactions with native plants are unlikely to change the conclusions, and we have attempted to compensate for this and any potential imbalance in the interaction data by also presenting the interactions in the functional trait space of plants. In addition to relying on known interactions, we evaluated whether it is theoretically possible for seeds to be swallowed.

### Analysis

Statistical analyses were done with R software v4.1.1 (R Development Core Team). An ANOVA was performed to compare the body mass distributions of the extinct, introduced and remaining frugivore groups (incl. birds, mammals and reptiles). Because the large introduced species reduced the normality of the body mass distribution (Supplementary Fig. 1), we also performed a non-parametric Kruskal–Wallis test. We compared a Tukey Honest Significant Differences post hoc test for the ANOVA with and without the two largest introduced species to determine whether they influenced the results (Supplementary Tables 1 and 2). All 40 frugivore species in the dataset were used for a linear regression between body mass and gape width. The interaction networks were visualized with the package bipartite and names of plants can be found in Supplementary Data 1 and 2. A principal coordinates analysis based on Gower distance (weighted 1/9 for fruit colors) was performed on the imputed plant trait dataset (Supplementary Fig. 2 and Supplementary Data 1). For each plant, the number of lost and gained interactions before and after extinctions and introductions was counted and categorized as having lost all interactions, lost some interactions, gained some interactions, or gained all known interactions. For each frugivore we calculated the 95% kernel density estimate of all plants that they are known to interact with (function kde, package ape^131^) and visualized these as circles in the functional trait space of the native plants. We compared the seed dispersers before and after extinctions and introductions, and we compared all extinct and introduced frugivores (including seed predators and those that only occasionally handle seeds).

### Reporting summary

Further information on research design is available in the Nature Portfolio Reporting Summary linked to this article.

## Supplementary information

Supplementary Information

Description of Additional Supplementary Files

Supplementary Data 1

Supplementary Data 2

Supplementary Data 3

Reporting Summary

## Data Availability

The plant species list and trait space data are provided in Supplementary Data 1 and 2. Information about the interactions is provided in Supplementary Data 3^13,34,42,44–46,48,51,52,71,81–126^.
