# Supplementary material for: Novel plant–frugivore network on Mauritius is unlikely to compensate for the extinction of seed dispersers
Source: Nat Commun. 2023 Feb 23;14:1019. doi: 10.1038/s41467-023-36669-9 (PMC9950440; doi:10.1038/s41467-023-36669-9)
Supplement: Supplementary file 1 — Supplementary Information [file 41467_2023_36669_MOESM1_ESM.pdf]

Supplementary information

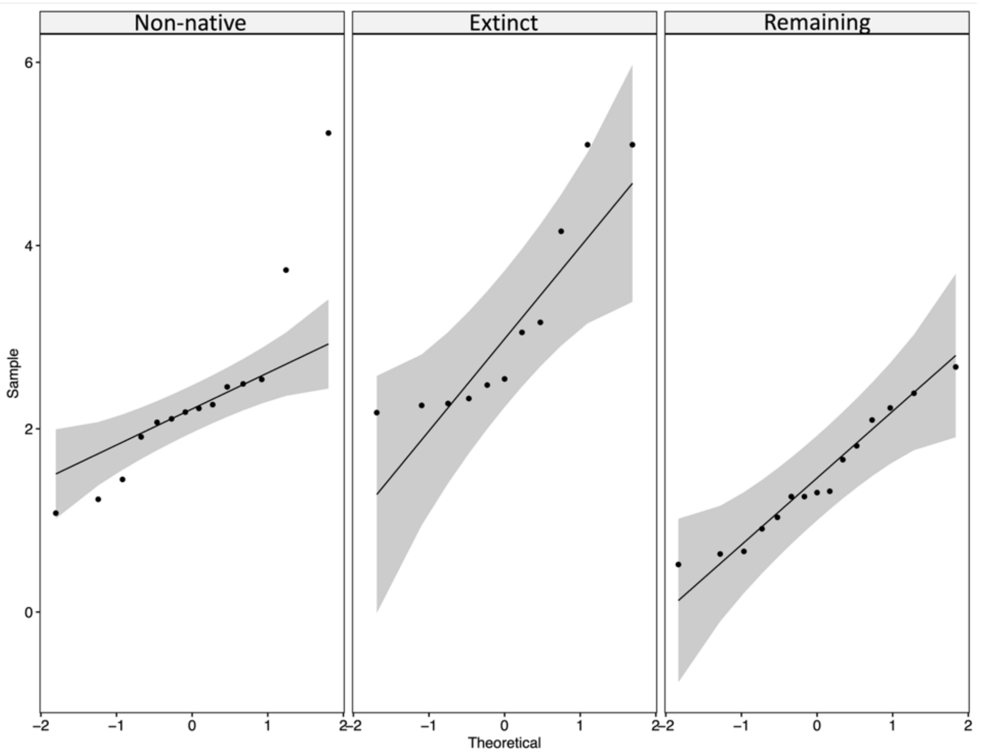

Figure 1. Normality of community body mass distributions. QQ plots that show normality for log_{10}-transformed body mass distributions of extinct, introduced and remaining frugivorous birds, mammals and reptiles on Mauritius (n = 40 species total, standard error in grey).

Table 1. Differences between community body mass distributions. Tukey post-hoc test for Honest Significant Differences on log_{10}-transformed body mass distributions of 11 extinct, 14 introduced and 15 remaining frugivorous vertebrate species (24 birds, 6 mammals and 10 reptiles) on Mauritius (n = 40 species total).

Group 1	Group 2	Estimate	Conf. low	Conf. high	p-value adj.	
Introduced	Extinct	0.794	-0.139	1.73	0.109	ns
Introduced	Extant	-0.904	-1.76	-0.0436	0.0377	*
Extinct	Extant	-1.70	-2.62	-0.778	0.000184	***

Table 2. Differences between community body mass distributions, without two large species. Tukey post-hoc test for Honest Significant Differences on log_{10}-transformed body mass distributions of 11 extinct, 14 introduced and 15 remaining frugivorous vertebrates (24 birds, 6 mammals and 10 reptiles) on Mauritius (n = 40 species total). Without two large introduced species (crab-eating macaque *Macaca fascicularis* 5.4 kg and feral pig *Sus scrofa* 169 kg).

Group 1	Group 2	Estimate	Conf. low	Conf. high	p-value adj.	
Introduced	Extinct	1.15	0.345	1.95	0.0036	**
Introduced	Extant	-0.550	-1.29	0.195	0.182	ns
Extinct	Extant	-1.70	-2.46	-0.934	0.0000123	****

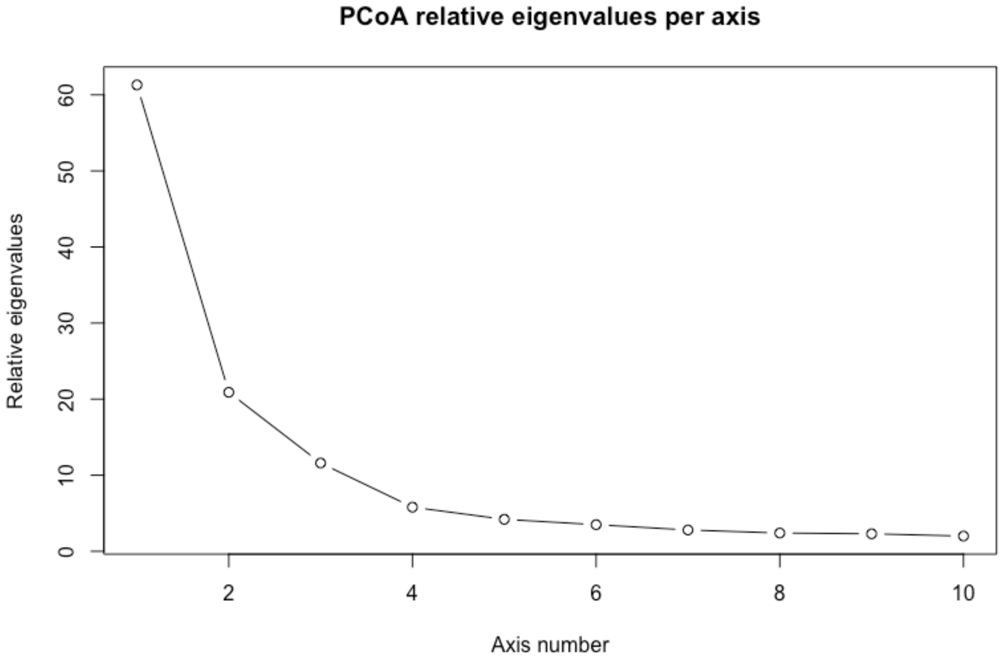

Figure 2. Explained variation by PCoA axis. PCoA relative eigenvalues per axis to show that most (82%) variation in the data is represented by the first two axes and 2D visualisation is appropriate.

Table 3. Trait imputation accuracy. Imputation of missing functional traits for plants with a random forest imputation method by R package missForest in preparation for PCoA. Average proportion of incorrectly classified data is 0.203488296. 25% of the plant trait database was imputed.

**Trait imputation with missForest**	
**Trait**	Mean Squared Error
Maximum fruit dimension	0.19053060
Minimum fruit dimension	0.19914060
Maximum seed dimension	0.06505226
Minimum seed dimension	0.08500232
Mean number of seeds	0.47771570

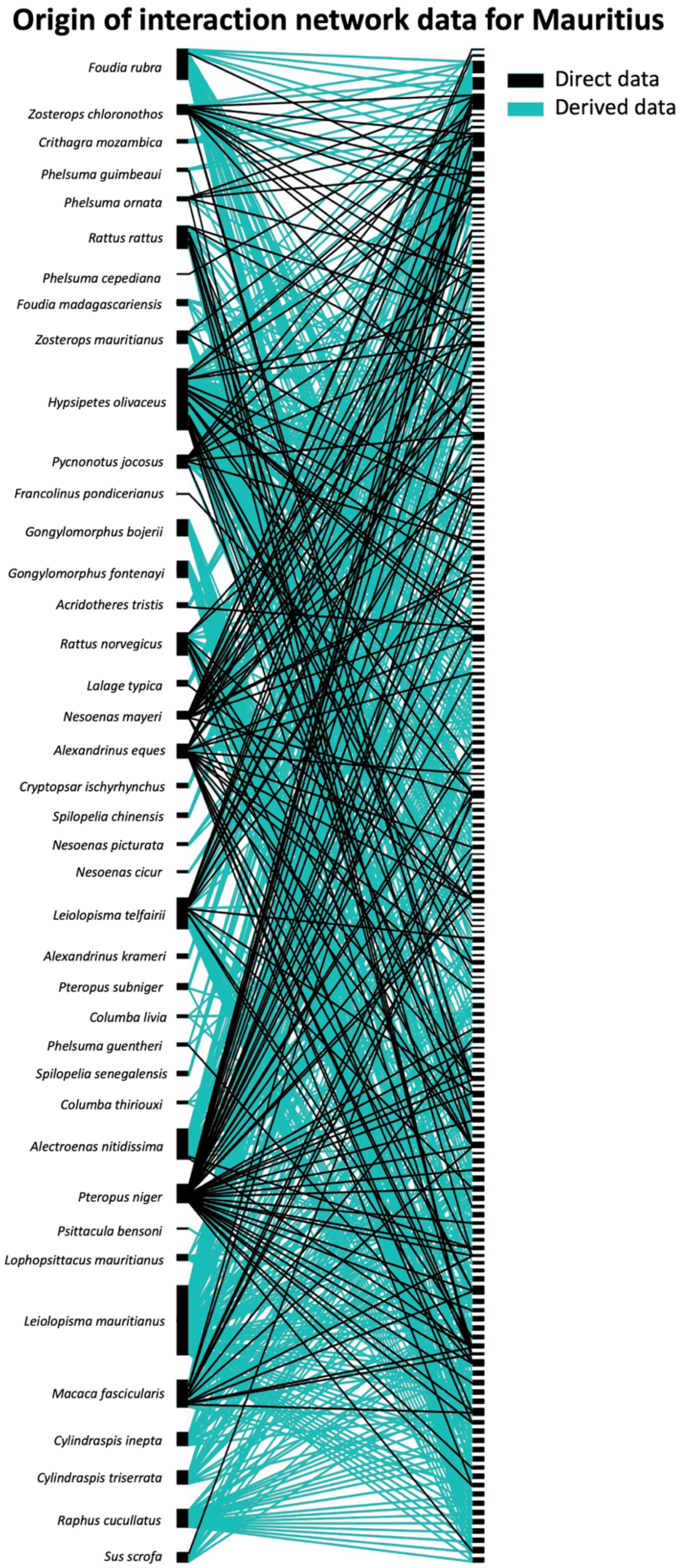

Figure 3. Interaction data origin. Direct data for the animal on Mauritius, or derived data from different locality or very closely related species in Indian Ocean region (e.g. Blue Pigeons in Seychelles). Plant names on the right can be found in Supplementary Data 2.

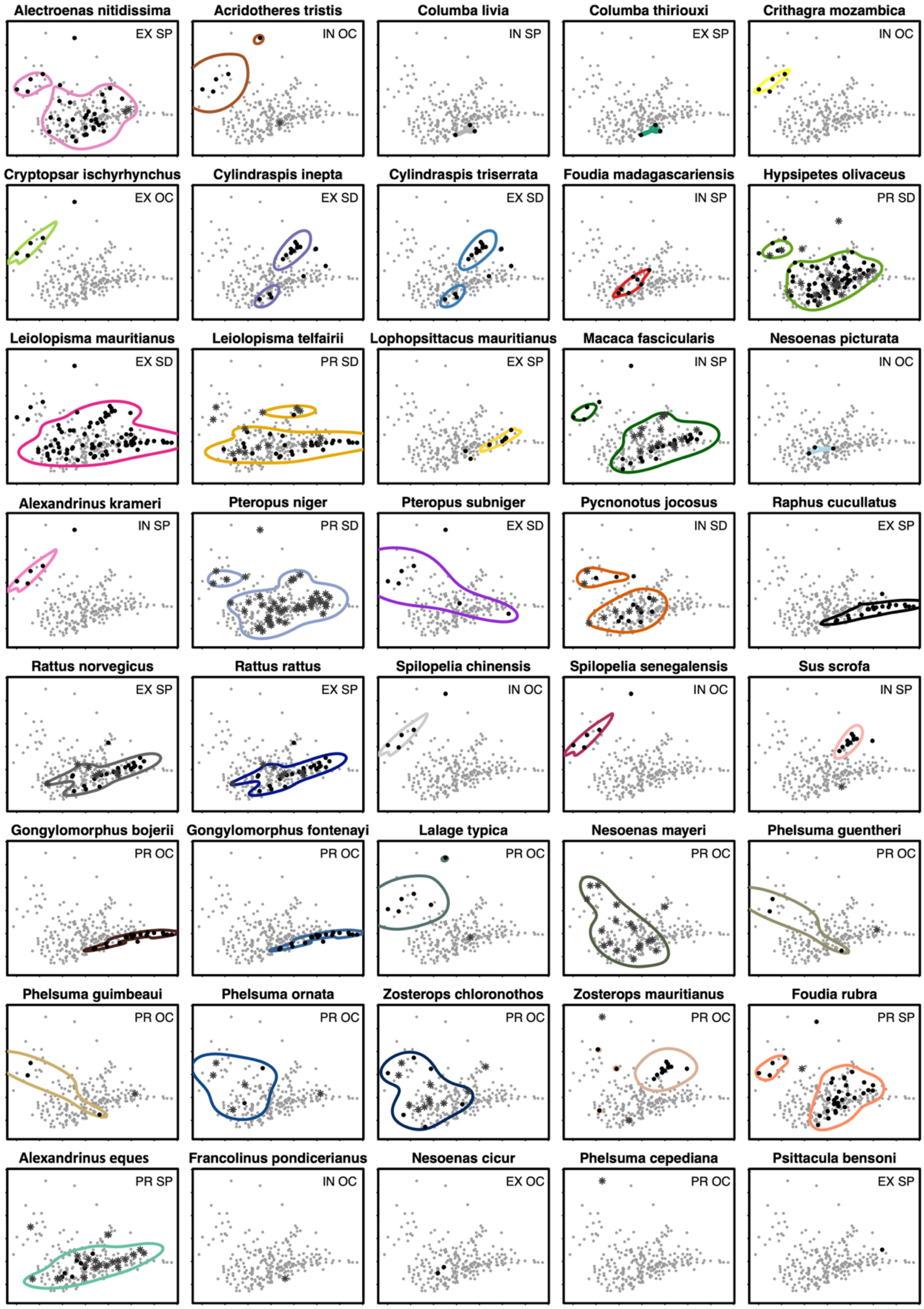

Figure 4. Individual diets of frugivores on Mauritius within the functional trait space of native plants. PCoA plots of plant functional traits (as in manuscript Figure 5) with 95% kernel densities of diet per frugivore species. Light grey points indicate native fleshy-fruited plant species occurring on Mauritius. Plant species included in the diet of the frugivore are indicated with black points when based on derived data and dark grey stars when based on direct data. EX=extinct, IN=introduced, PR=present, SD=seed disperser, SP=seed predator, OC=occasional frugivore. Plant names in Supplementary Data 1.

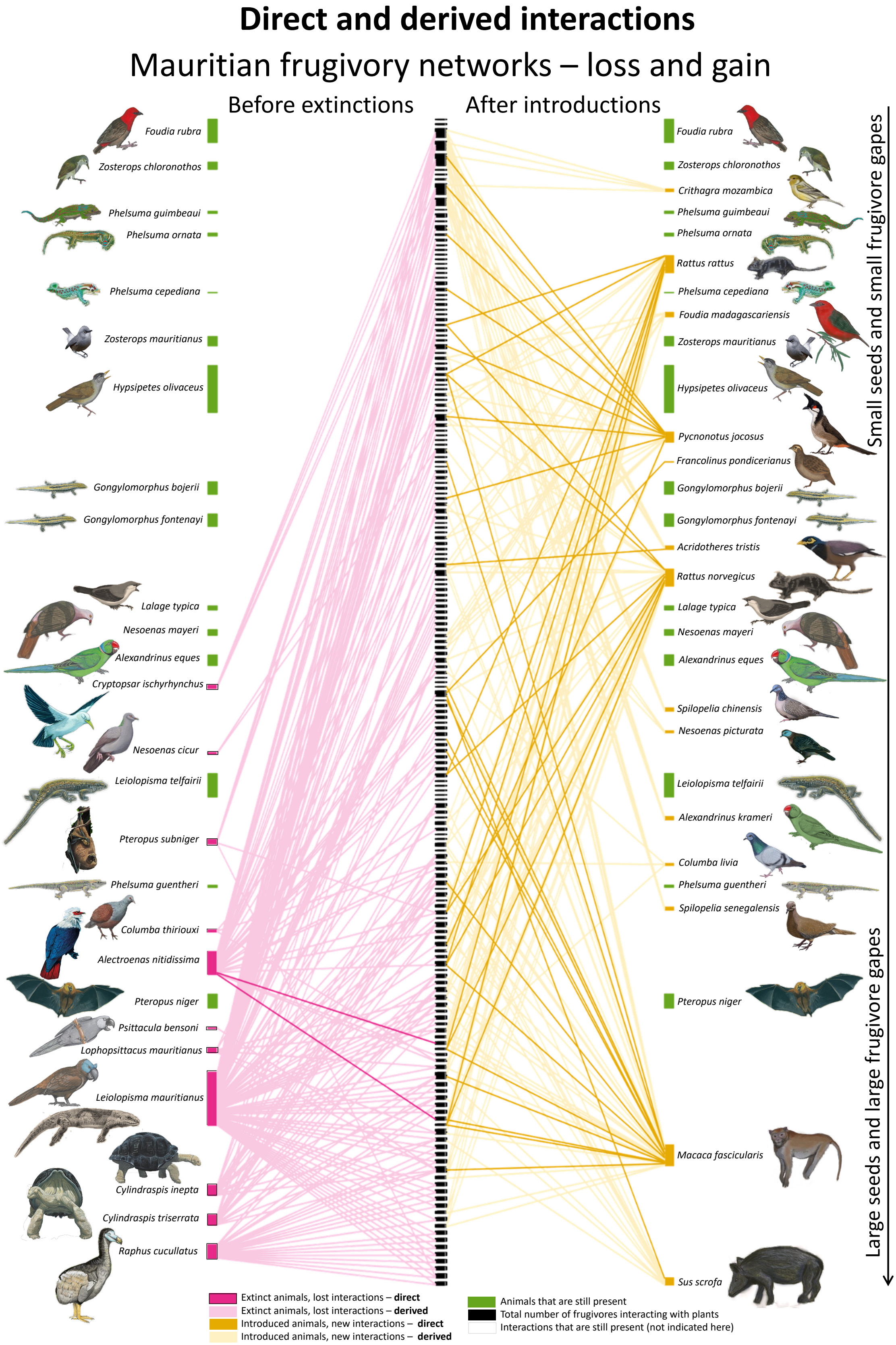

Figure 5. **Plant-frugivore networks with distinction between direct and derived interaction data origin.** Interaction network between frugivores and native fleshy-fruited plants on Mauritius, before and after extinctions and introductions. Pink lines indicate lost interactions and yellow lines indicate gained interactions. Interactions with remaining frugivores have been left out here for readability. Plant names in Supplementary Data 2. Drawings by JPH and JHH.

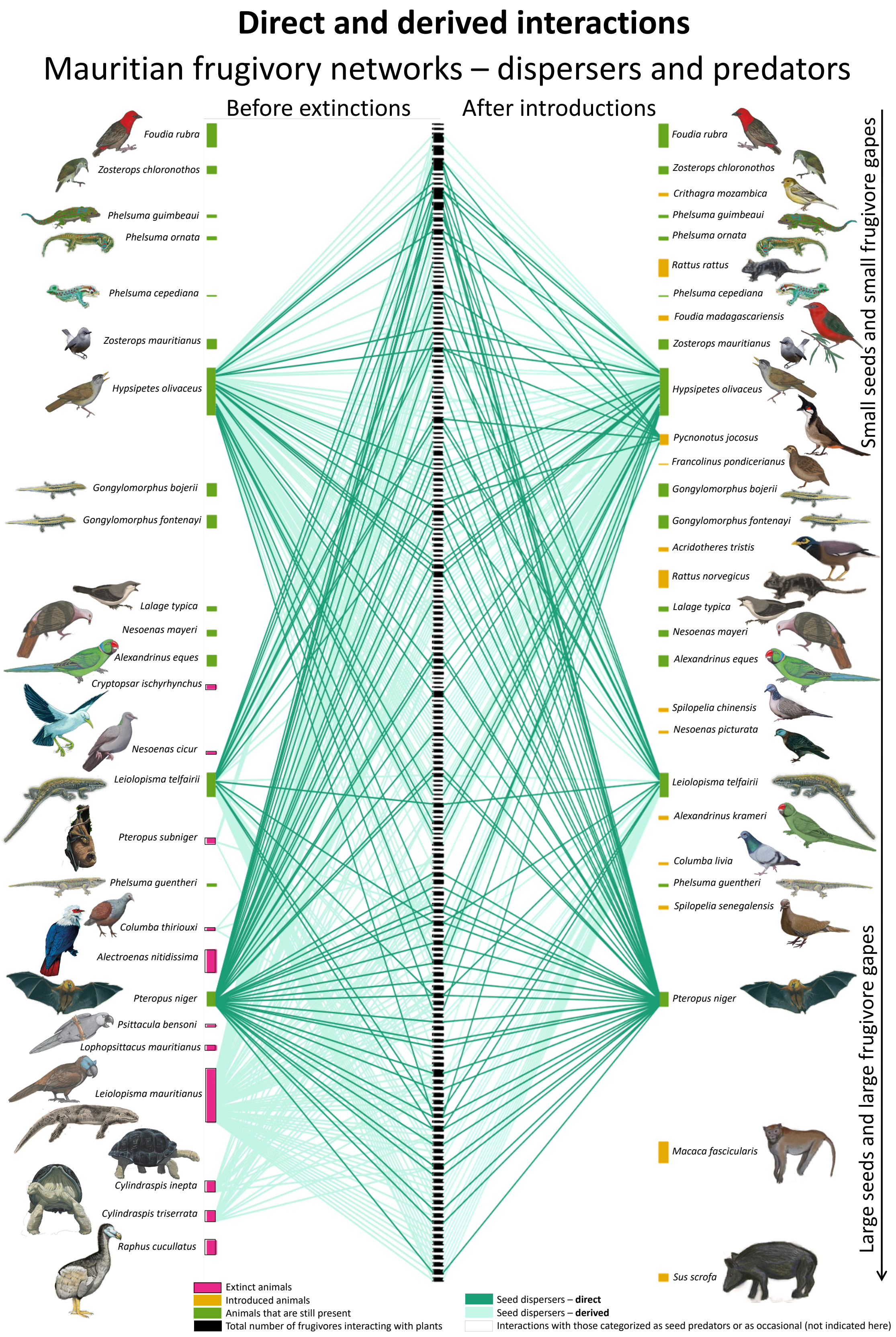

Figure 6. **Seed disperser networks with distinction between direct and derived interaction data origin.** Interaction network between frugivores and native fleshy-fruited plants on Mauritius, before and after extinctions and introductions. Green lines indicate interactions with seed dispersers. Interactions with those that are not categorized as seed dispersers have been left out here for readability. Plant names in Supplementary Data 2. Drawings by JPH and JHH.
