# Supplementary material for: Novel plant–frugivore network on Mauritius is unlikely to compensate for the extinction of seed dispersers
Source: Nat Commun. 2023 Feb 23;14:1019. doi: 10.1038/s41467-023-36669-9 (PMC9950440; doi:10.1038/s41467-023-36669-9)
Supplement: Supplementary file 2 — Description of Additional Supplementary Files [file 41467_2023_36669_MOESM2_ESM.pdf]

Description of Additional Supplementary Files

File Name: **Supplementary Data 1**
Description: **All native Mauritian fleshy-fruited plant species.**
Names of all 263 native fleshy-fruited plant species on Mauritius for which there is at least one trait known, sorted according to maximum seed dimension as in Manuscript Figure 2 and PCoA axis of plant traits (maximum and minimum fruit and seed sizes, number of seeds per fruit and fruit colour) as in Manuscript Figures 5 and 6, and Supplementary Figure 4. Percentage of original data indicates traits that are not imputed with missForest or, in case of fruit colour, filled in with genus or family average.

File Name: **Supplementary Data 2**
Description: **Native Mauritian fleshy-fruited plant species with known frugivore interactions.** Names of 191 native fleshy-fruited plants on Mauritius that have known interactions with frugivores (directly observed or derived data), sorted according to maximum seed dimension, as in the networks in Manuscript Figures 3 and 4, and Supplementary Figures 3, 5 and 6. Plant species that have imputed values for this trait are indicated in bold and in the last column.

File Name: **Supplementary Data 3**
Description: **Literature-based justifications of diet and seed handling categorization for vertebrates on Mauritius**.
