# Supplementary material for: Novel plant–frugivore network on Mauritius is unlikely to compensate for the extinction of seed dispersers
Source: Nat Commun. 2023 Feb 23;14:1019. doi: 10.1038/s41467-023-36669-9 (PMC9950440; doi:10.1038/s41467-023-36669-9)
Supplement: Supplementary file 6 — Reporting Summary [file 41467_2023_36669_MOESM6_ESM.pdf]

nature portfolio

Corresponding author(s): Julia Helena Heinen

Last updated by author(s): Feb 4, 2023

Reporting Summary

Nature Portfolio wishes to improve the reproducibility of the work that we publish. This form provides structure for consistency and transparency in reporting. For further information on Nature Portfolio policies, see our Editorial Policies and the Editorial Policy Checklist.

Statistics

For all statistical analyses, confirm that the following items are present in the figure legend, table legend, main text, or Methods section.

n/a	Confirmed	
☐	☒	The exact sample size (*n*) for each experimental group/condition, given as a discrete number and unit of measurement
☒	☐	A statement on whether measurements were taken from distinct samples or whether the same sample was measured repeatedly
☐	☒	The statistical test(s) used AND whether they are one- or two-sided<br>*Only common tests should be described solely by name; describe more complex techniques in the Methods section.*
☒	☐	A description of all covariates tested
☐	☒	A description of any assumptions or corrections, such as tests of normality and adjustment for multiple comparisons
☐	☒	A full description of the statistical parameters including central tendency (e.g. means) or other basic estimates (e.g. regression coefficient) AND variation (e.g. standard deviation) or associated estimates of uncertainty (e.g. confidence intervals)
☐	☒	For null hypothesis testing, the test statistic (e.g. *F*, *t*, *r*) with confidence intervals, effect sizes, degrees of freedom and *P* value noted<br>*Give P values as exact values whenever suitable.*
☒	☐	For Bayesian analysis, information on the choice of priors and Markov chain Monte Carlo settings
☒	☐	For hierarchical and complex designs, identification of the appropriate level for tests and full reporting of outcomes
☒	☐	Estimates of effect sizes (e.g. Cohen's *d*, Pearson's *r*), indicating how they were calculated

Our web collection on statistics for biologists contains articles on many of the points above.

Software and code

Policy information about availability of computer code

Data collection	No software was used.
Data analysis	R Software v4.1.1, packages ape, bipartite, missForest, PVR, v.PhyloMaker.

For manuscripts utilizing custom algorithms or software that are central to the research but not yet described in published literature, software must be made available to editors and reviewers. We strongly encourage code deposition in a community repository (e.g. GitHub). See the Nature Portfolio guidelines for submitting code & software for further information.

Data

Policy information about availability of data

All manuscripts must include a data availability statement. This statement should provide the following information, where applicable:

- Accession codes, unique identifiers, or web links for publicly available datasets
- A description of any restrictions on data availability
- For clinical datasets or third party data, please ensure that the statement adheres to our policy

The databases were made for this study. The plant species list and trait space data are provided in Supplementary Data 1 and 2. Information about the interactions is provided in Supplementary Data 3

Human research participants

Policy information about studies involving human research participants and Sex and Gender in Research.

Reporting on sex and gender	No research on humans was done.
Population characteristics	No research on humans was done.
Recruitment	No research on humans was done.
Ethics oversight	No ethics approval was needed.

Note that full information on the approval of the study protocol must also be provided in the manuscript.

Field-specific reporting

Please select the one below that is the best fit for your research. If you are not sure, read the appropriate sections before making your selection.

☐ Life sciences ☐ Behavioural & social sciences ☒ Ecological, evolutionary & environmental sciences

For a reference copy of the document with all sections, see nature.com/documents/nr-reporting-summary-flat.pdf

Ecological, evolutionary & environmental sciences study design

All studies must disclose on these points even when the disclosure is negative.

Study description	Species-level data was collected (literature, herbariums, museums, fresh plants) on all native Mauritian fleshy-fruited plants (fruit and seed sizes and color), all fruit-eating animal species occurring on Mauritius (native, extinct and introduced birds, mammals and reptiles) (jaw width and body mass). Plant-animal interaction data was collected from literature.
Research sample	Fruit and seed sizes, fruit colour and number of seeds of all native Mauritian fleshy-fruited plant species. Body mass, gape size and native fruits known from the diet of all fruit-eating animal species occurring on Mauritius (native, extinct and introduced birds, mammals and reptiles). Data and sources can be found in Supplementary Data 1, 2 and 3.
Sampling strategy	Species-level information was used. When multiple specimens were available, average measurements of all available specimens were used.
Data collection	Data was collected from literature and measurements in the field and herbaria and museum collections (CB, FBVF, JHH, JPH). Interaction data between plants and animals was found with google scholar searches for the animal name and "fruit" or "berry". For native fleshy-fruited plants, fruit color was determined as basic color terms during field observations and from literature descriptions (CB, FBVF, JHH). Measurements of maximum fruit and seed dimensions were taken from fresh fruits or re-hydrated fruits in herbaria (CB, FBVF, JHH) with calipers. Animal body mass data was obtained from literature or obtained according to the calculations based on other morphometrics used by Heinen et al. 2018, Ecography. Animal skins and skeletons (JHH, JPH) were used to obtain gape size data as distance between commisural points, with calipers.
Timing and spatial scale	Data used in this study was collected over the past four centuries in the Indian Ocean region because this is the time humans have been present on the island and species have been introduced and have gone extinct. Data are from all areas of the island Mauritius and the surrounding islets.
Data exclusions	No data was excluded.
Reproducibility	No experiments were performed.
Randomization	No experiments were performed.
Blinding	No experiments were performed.

Did the study involve field work? ☒ Yes ☐ No

Field work, collection and transport

Field conditions	Data collected over the past four centuries in Mauritius during various field conditions.

Location	Mauritius
Access & import/export	Fruits and seeds measured in Mauritius under permit NP46V8, issued on 2 May 2017 by the National Parks and Conservation Services (with permission from government) in Mauritius. Other measurements taken in herbaria and museums. No samples were exported.
Disturbance	No disturbance was caused by the study.

Reporting for specific materials, systems and methods

We require information from authors about some types of materials, experimental systems and methods used in many studies. Here, indicate whether each material, system or method listed is relevant to your study. If you are not sure if a list item applies to your research, read the appropriate section before selecting a response.

Materials & experimental systems

n/a	Involved in the study
☒	☐ Antibodies
☒	☐ Eukaryotic cell lines
☒	☐ Palaeontology and archaeology
☐	☒ Animals and other organisms
☒	☐ Clinical data
☒	☐ Dual use research of concern

Methods

n/a	Involved in the study
☒	☐ ChIP-seq
☒	☐ Flow cytometry
☒	☐ MRI-based neuroimaging

Animals and other research organisms

Policy information about studies involving animals; ARRIVE guidelines recommended for reporting animal research, and Sex and Gender in Research

Laboratory animals	None used.
Wild animals	None used.
Reporting on sex	All available animal specimens in museum collections were used and average values calculated per species, across sexes.
Field-collected samples	No samples were collected.
Ethics oversight	Permit for measuring fruits and seeds was issued by the government of Mauritius that had no ethical concerns.

Note that full information on the approval of the study protocol must also be provided in the manuscript.
